# Outpatient management of large scalp aplasia cutis congenita without skull defect in a case of Adams‐Oliver syndrome

**DOI:** 10.1002/kjm2.12935

**Published:** 2025-01-23

**Authors:** Yu‐Ting Tsai, Yi‐Chien Yang

**Affiliations:** ^1^ Department of Dermatology Kaoshiung Chang Gung Memorial Hospital Kaohsiung City Taiwan

Dear Editor,

Adams‐Oliver syndrome (AOS) is a genetic disorder characterized by aplasia cutis congenita (ACC), a condition involving the absence of skin, including the epidermis, dermis, and sometimes subcutaneous tissue, primarily affecting the scalp. This condition is frequently associated with terminal transverse limb defects and abnormalities in the cardiac and central nervous systems.^1^ Recent advances in wound care have shifted the management of large ACC lesions toward conservative approaches.^2^ However, data on the effectiveness of these conservative approaches in AOS patients remains limited. This report details the management of a large scalp ACC without skull defect in an infant with AOS using outpatient wound care, supported by serial follow‐up photographs.

The patient, a male newborn, was delivered via cesarean section at 38 weeks of gestation to healthy, non‐consanguineous parents. He presented with a large, irregularly shaped scalp defect extending to the subcutis, without underlying bony involvement, measuring approximately 8 cm × 5 cm. The lesion, primarily affecting the midline frontal, parietal, and occipital regions, showed ulceration and necrosis (Figure 1A). Additional findings included cutis marmorata telangiectatica congenita, hypoplastic toenails, and syndactyly of the right second, third, and fourth toes. Echocardiography revealed a patent foramen ovale and a patent ductus arteriosus, while brain ultrasound showed no structural abnormalities. Other physical, neurological, respiratory, gastrointestinal, and genitourinary examinations were normal. The patient was diagnosed with AOS and type 2 scalp ACC. Genetic testing was not performed due to lack of parental consent.

**FIGURE 1:**
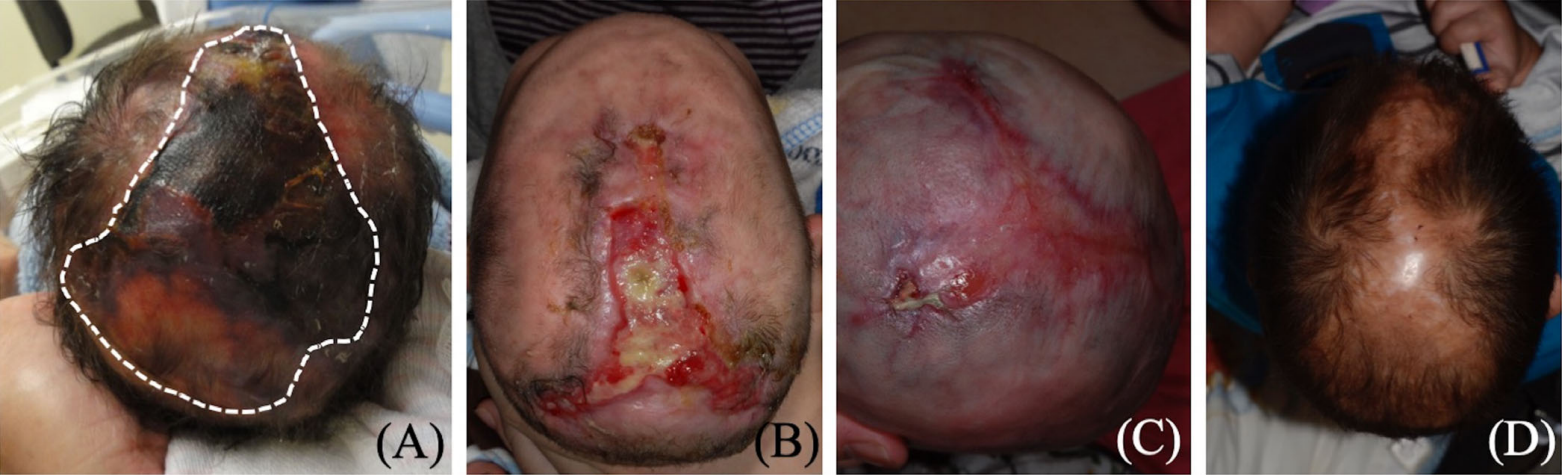
Serial follow‐up of the scalp aplastic cutis in the Adams‐Oliver syndrome patient at (A) 1 day old, (B) 2 months old, (C) 4 months old, and (D) 2 years old.

The neurosurgery and plastic surgery teams recommended a conservative approach for the ACC. The discharge planning team opted for hydrogel and foam wound dressings, chosen for their efficacy in rehydrating the wound, facilitating necrotic tissue removal, and promoting autolytic debridement. These non‐adherent dressings were advantageous in protecting fragile tissue and simplifying at‐home dressing changes for the caregivers. The family received training on wound care, and the patient was discharged with a plan for weekly outpatient visits for 3 months, later reduced to biweekly visits.

During the 6‐month healing period, the patient developed a *Pseudomonas aeruginosa* infection in the second month (Figure 1B), which was successfully treated with topical gentamicin cream and oral ciprofloxacin for 2 weeks. After resolving the infection and hydrolyzing the eschar, silver‐containing silicone foam dressings were used to prevent reinfection. By the fourth month, the scalp defect had nearly fully epithelialized without complications (Figure 1C). The patent foramen ovale and ductus arteriosus closed spontaneously by 1 year of age. The patient subsequently underwent staged surgery to separate the syndactyly. At the 2‐year follow‐up, no significant medical issues were noted, though a healed scar with alopecia remained (Figure 1D).

The healing trajectory of ACC in AOS generally aligns with typical wound‐healing patterns. However, genetic factors in AOS may disrupt vascular development and cellular migration, potentially delaying healing, particularly in larger defects or areas with poor vascular supply.^3^ While natural healing likely contributed to this patient's improvement, the conservative wound management regimen facilitated closure and mitigated complications. This case demonstrates that advanced outpatient wound management with hydrogel and foam dressings can effectively treat large scalp ACC in AOS patients, reducing the need for hospitalization. Conservative wound care, combined with vigilant infection monitoring, is a viable strategy for large scalp defects without bony involvement. For cases involving skull defects, multidisciplinary management may be necessary, as rare complications may require additional interventions.^4^

## CONFLICT OF INTEREST STATEMENT

All authors declare no conflicts of interest.

## Data Availability

The data that support the findings of this study are available from the corresponding author upon reasonable request.
